# Resistin in Human Seminal Plasma: Relationship with Lipid Peroxidation, CAT Activity, GSH/GSSG Ratio, and Semen Parameters

**DOI:** 10.1155/2019/2192093

**Published:** 2019-10-22

**Authors:** Elena Moretti, Lucia Micheli, Daria Noto, Anna Ida Fiaschi, Andrea Menchiari, Daniela Cerretani

**Affiliations:** ^1^Department of Molecular and Developmental Medicine, University of Siena, 53100 Siena, Italy; ^2^Department of Medical and Surgical Sciences and Neurosciences, University of Siena, 53100 Siena, Italy; ^3^Department of Business and Law, University of Siena, 53100 Siena, Italy

## Abstract

Resistin is an adipokine involved in inflammation and able to induce the expression of other proinflammatory cytokines. It is known that, in human semen, resistin is correlated with inflammatory cytokines and sperm quality. The aim of this prospective study was to explore the potential relationship between resistin, lipid peroxidation (LPO), catalase (CAT) activity, and reduced and oxidized glutathione (GSH/GSSG) ratio in semen samples of infertile patients with leukocytospermia (no. 19), infertile patients with varicocele (no. 17), and fertile men (no. 17). Semen analysis was performed following the WHO guidelines, and sperm apoptosis and necrosis were evaluated with annexin V/propidium iodide assay. Seminal plasma samples were used to determine resistin levels by an immunological method, MDA concentration by a HPLC analysis with UV detection, GSH/GSSG ratio by an enzymatic method, CAT activity by a spectrophotometric method. The results showed that, in both groups of infertile patients, semen parameters were significantly reduced (*P* < 0.001) and sperm apoptosis and necrosis percentages were increased. Resistin levels were significantly higher in leukocytospermia and varicocele groups (*P* < 0.001 and *P* < 0.01, respectively) as well as MDA concentration (*P* < 0.001) compared to controls. The MDA level was also significantly increased in the leukocytospermia group versus the varicocele group (*P* < 0.05). The GSH/GSSG ratio was higher in fertile controls than the leukocytospermia group (*P* < 0.05) and the varicocele group (*P* < 0.001) and in the leukocytospermia group versus the varicocele group (*P* < 0.05). Both the leukocytospermia and varicocele groups showed increased values of CAT activities (*P* < 0.001) than controls. Briefly, the correlation between variables, calculated in the whole patient population, showed that resistin levels positively correlated with MDA levels, CAT activity, sperm apoptosis, and necrosis and negatively with sperm parameters and GSH/GSSG ratio. These results support an active role of resistin in an inflammatory process causing LPO, increase of CAT activity, and decrease of GSH/GSSG ratio in seminal plasma of infertile men vs. fertile controls.

## 1. Introduction

Adipokines, such as leptin, resistin, adiponectin, chemerin, omentin, and visfatin, play a critical role in the development of complications related to obesity and inflammatory conditions [1–3]. In addition, they are also involved in other functions of the organism including those pertinent to the gonadal and hypothalamic-pituitary axis, both in females and in males. Among these adipokines, we studied resistin, a cytokine that belongs to a family of low molecular weight cysteine-rich secretory proteins, synthesized by adipose tissue. It is well known that resistin regulates glucose metabolism in mammalians and that high levels of circulating resistin are responsible for insulin resistance [4]. Therefore, it is postulated that resistin represents a molecular link between obesity and type 2 diabetes [5]. Patel et al. [6] demonstrated that, in contrast to what it was observed in mouse, resistin was almost undetectable in human adipose tissue. In addition, the analysis of the resistin gene expression in different human tissues revealed that macrophages, peripheral blood mononuclear cells, and bone marrow represent the main sources of resistin [7–10]. These studies demonstrated the role of resistin in inflammatory pathways since proinflammatory mediators are able to enhance resistin expression in peripheral mononuclear cells [11]. Similarly to the other adipokines, resistin is involved in both male and female reproductive functions [2]. With regard specifically to the male reproductive system, the expression of resistin is controlled by gonadotropins, indicating that this peptide plays a hormonal impact upon the testes [9]. Despite resistin was found in rat Leydig and Sertoli cells within the seminiferous tubules [12], resistin human testis localization has not been explored yet. A recent review dealing with adipokines in semen [13] reported that only three studies measured resistin levels in human seminal plasma. Some of our group found that semen resistin levels negatively correlated with sperm motility and viability and positively with sperm apoptosis and necrosis [14]. These data were not in accord with those reported by Kratzsch et al. [15] and Thomas et al. [16]. Despite these differences, the authors of both groups [14, 15] agree on the positive relationship between semen resistin concentrations and semen levels of proinflammatory mediators such as elastase, interleukin-6 (IL-6), and tumour necrosis factor-alpha (TNF-alpha) [14, 15]. Such observation may suggest a potential role of resistin as a marker of inflammation in human semen. Indeed, in the course of inflammatory events or under other pathological conditions, cytokine levels and reactive oxygen species (ROS) increase. In particular, ROS are necessary in several physiological steps such as development and maturation of spermatozoa, capacitation, acrosome reaction, and fertilization [17]. When, in seminal plasma, an imbalance between oxidants and antioxidants in favor of the antioxidants occurs [18], a disruption of redox signaling and control can interfere with normal sperm function causing membrane lipid peroxidation (LPO), DNA fragmentation, etc. [19, 20]. It was recently demonstrated that induced varicocele has a negative effect on rat spermatogenesis and increases oxidative stress leading to the production of sperm with damaged chromatin which reduces the fertility potential [21]. Since a relationship between resistin and oxidative stress in pathologies such as diabetes mellitus [3, 22] and fatty liver disease [1] was reported, we decided to study this subject in human semen. Thus, we determined resistin levels in seminal plasma of infertile patients affected by leukocytospermia or varicocele and in a group of fertile individuals. In the same samples, the LPO and redox imbalance were assessed by malondialdehyde (MDA) and antioxidant processes, such as those related to reduced and oxidized glutathione (GSH/GSSG) ratio and catalase (CAT) activity. In human semen, the relationship between these parameters was still unexplored; therefore, this research represents a new subject of study.

## 2. Materials and Methods

### 2.1. Patients and Controls

Among patients attending our laboratory for semen analysis, we selected 36 consecutive infertile patients (aged 25-35) with leukocytospermia or varicocele. Infertile patients did not obtain pregnancy after two years of unprotected sexual intercourses; the female factor infertility was excluded. 19 patients (aged 27-35) showed leukocytospermia and 17 patients (aged 25-32) varicocele. Leukocytospermia was determined during semen analysis following the WHO guidelines [23]. Varicocele was assessed by both physical examination and scrotal colour Doppler ultrasonography analysis performed in laboratories different from ours. For this study, we included patients with grade II and grade III varicocele.

All patients satisfied the following criteria: non-azoospermic men, 46, XY karyotype, BMI < 25 kg/m^2^. They showed normal levels of follicle-stimulating hormone (FSH), luteinizing hormone (LH), and testosterone (T). Hormone concentrations were determined in serum by commercial kits (Beckman Coulter Access for FSH, LH, and testosterone). A normal range for FSH was 0.7-11.00 mU/ml (sensitivity 0.2 mUI/ml, intra- and interassay coefficient of variation < 10%), for LH was 0.8-8.0 mU/l (sensitivity 0.2 mUI/ml, intra- and interassay coefficient of variation < 10%), and for T was 2.7-10.9 mg/ml (sensitivity 0.1 ng/ml, intra- and interassay coefficient of variation < 10%). Genitourinary infections were excluded by semen culture. Samples were seeded using a calibrated loop on agar plates, which were incubated overnight at 37°C in normal air with 5% CO_2_. The microorganisms were identified by gram stain, oxidase, catalase, and other biochemical tests using BioMérieux products (BioMérieux, Florence, Italy). Semen cultures were positive when the number of colonies was ≥10^4^ CFU ml^−1^ in case of gram positive cocci and ≥10^5^ CFU ml^−1^ in case of gram negative rods. The selected patients which had no chronic diseases and did not receive medication, chemotherapy, and radiotherapy. None of subjects took oral antioxidant supplements for at least five months before the analysis. Subjects with a history of recreational drug use, alcohol consumption, and smoking habit were excluded from this study.

17 fertile men (aged 25-33) which fathered at least one child in the last 3 years represented the control group. These subjects were not affected by infections and anatomical and hormonal problems. Their semen parameters were higher than 25 percentiles as reported in the WHO guidelines [23].

Before the inclusion in this study, all patients and controls provided an informed written consent. The informed consent describes the aims of the research, and it is approved by the Institutional Review Board of Siena University.

### 2.2. Semen Analysis

The analysis of human semen samples was performed following the WHO guidelines [23]. Samples were collected by masturbation after 3-5 days of sexual abstinence and analysed after liquefaction for 30 min at 37°C. Semen volume, pH, sperm concentration, and motility were determined. The Papanicolaou (PAP) test modified for spermatozoa enabled to assess sperm morphology. Leukocytes in semen samples were identified by peroxidase stain, and a leukocyte concentration ≥ 1 million cell/ml was considered abnormal (WHO, [23]).

### 2.3. Detection of Sperm Apoptosis and Necrosis

To detect sperm apoptosis and necrosis, the Vybrant apoptosis assay (Invitrogen Ltd., Paisley, United Kingdom) based on fluorescein isothiocyanate- (FITC-) annexin V (AnV, green fluorescence) and propidium iodide (PI, red fluorescence) was used. AnV protein binds to negatively charged phospholipid phosphatidylserine, located in the inner leaflet of the plasma membrane, that during apoptosis is actively externalized to the outer leaflet of the plasma membrane. PI stains necrotic cell with broken membrane. The detailed procedure is described in Moretti et al. [14]. About 300 sperms for each sample were scored, and undamaged sperm (AnV negative, PI negative, not stained), apoptotic sperm (AnV positive, PI negative, green stained), and necrotic sperm (AnV negative, PI positive, red stained) were classified. Sperms showing both green and red signals were considered as necrotic since their membrane was damaged.

### 2.4. Resistin Assay

Resistin was determined in semen samples of the 53 participants to the study. 500 *μ*l of each semen sample was collected 1 h after production and fractioned by centrifugation (200 g for 15 min). The supernatant, composed of seminal plasma without spermatozoa, was stored at -80°C until resistin levels were evaluated by enzyme-linked immunosorbent assay (ELISA) using resistin (human) ELISA kit (Phoenix Pharmaceutical, Inc., Burlingame, CA, USA), following the manufacturer's instructions. Briefly, samples were added to microtiter wells sensitized with anti-human resistin antibodies. Some wells contained known amounts of resistin, which are important to build the regression line. After incubation for 2 hours at room temperature, wells were washed. Then, wells were incubated with the biotinylated anti-human resistin detection antibody diluted 1 : 150 for 2 hours at room temperature. At the end of incubation, wells were washed and streptavidin-horseradish peroxidase solution was added into each well and incubated for 30 min at room temperature. After washing, the substrate solution provided in the kit was added into each well; finally, the reaction was stopped with 2N hydrochloric acid. The optical density was read at 450 nm using the iMark™ Microplate Absorbance Reader (Bio-Rad, Italy). The results were expressed in ng/ml.

### 2.5. Reduced and Oxidized Glutathione Evaluation

After thawing, 200 *μ*l of seminal plasma without spermatozoa of each subject was diluted with an equal volume of 10% metaphosphoric acid. Specimens were centrifuged at 2000 g for 10 min at 0°C. Total glutathione (GSH) and oxidized glutathione (GSSG) were measured in the supernatant by a microassay method [24] and expressed in nmol per mg of protein. Each sample was determined in triplicate, and these indices were expressed as reduced (GSH) to oxidized glutathione (GSSG) ratio.

### 2.6. Catalase Activity Determination

After thawing, seminal plasma without spermatozoa of each subject was centrifuged at 4000 g for 15 min at 4°C. A microassay procedure was used [25] to determine the catalase (CAT) activity.

This method that requires 20 *μ*l of seminal plasma depends on the reaction of CAT with methanol in the presence of an optimal concentration of hydrogen peroxide. The formaldehyde production was measured spectrophotometrically at 540 nm with 4-amino-3-hydrazino-5-mercapto-1,2,4-triazole (Purpald, Sigma-Aldrich, Milan, Italy) as a chromogen. One unit of catalase activity was defined as the amount of enzyme that caused the formation of 1 nmol of formaldehyde per min at 25°C. The readings were made three times (20 *μ*l each time). Results were expressed as nmol/min/mg of protein.

### 2.7. Protein Assay

Protein concentrations were determined according to Lowry et al., and the calibration curves were prepared with dry bovine serum albumin [26].

### 2.8. Malondialdehyde (MDA) Assessment

Free MDA levels were measured in seminal plasma without spermatozoa. After thawing, 500 *μ*l of seminal plasma was added to 500 *μ*l of tris-HCl 0.04 M and acetonitrile containing 0.1% butylated hydroxytoluene (BHT) to prevent artifact oxidation of polyunsaturated free fatty acids during the assay. The samples were centrifuged at 3000 g for 15 min. The supernatant was used for MDA HPLC analysis with UV detection after derivatization with 2·4 dinitrophenylhydrazine according the method of Shara et al. [27] with minor modifications. The samples were immediately stirred and were extracted with 5 ml of pentane; finally, the samples were dried by using nitrogen. A calibration with concentrations of MDA by 0.5 nmoli/ml to 10 nmoli/ml was used for MDA determinations. The MDA hydrazone was quantified by isocratic high-performance liquid chromatography using a Waters 600 E System Controller HPLC (Milford, MA, USA) equipped with a Waters Dual *λ* 2487 UV detector (Milford, MA, USA) set at 307 nm. To separate the hydrazone derivative, a 5 *μ* ultrasphere ODS column C18 (Beckman, San Ramon, CA, USA) was used at the flow rate of 0.8 ml/min with the acetonitrile (45%)-HCl 0.01 N (55%) as mobile phase. The concentrations of MDA were obtained by peak areas determined using an Agilent 3395 integrator (Agilent Technologies, USA). Each sample was assessed in triplicate, and the results are expressed in nmol of MDA per ml of seminal plasma.

### 2.9. Statistical Analysis

Statistical analysis was performed using the SPSS software package (version 19, SPSS Inc., Chicago, IL, USA). The Kolmogorov-Smirnov test was used in order to examine the normality of distribution of the variables investigated. Levene's test were used to verify the assumption of homoscedasticity of the variance of groups. One-way analysis of variance (ANOVA) was utilized to evaluate differences among the groups. When the assumption of homoscedasticity was respected, Tukey's test was used for multiple comparisons. The Welch test and Games-Howell post hoc test were used in condition of heteroskedasticity. The values of *P* < 0.05 were considered significant. The correlation between the investigated variables was assessed using Spearman's rank correlation coefficient (rho).

## 3. Results

The semen variables of the 53 subjects grouped as infertile patients with leukocytospermia (no. 19), infertile patients with varicocele (no. 17), and fertile controls (no. 17) are shown in Table 1. The values of semen volume were similar in the three groups; the sperm concentration, the percentage of progressive motility, and the percentage of sperm with normal morphology were significantly reduced in both groups of infertile patients (*P* < 0.001) compared to those observed in the fertile group (Table 1). The percentage of apoptotic sperm was significantly higher in the leukocytospermia and varicocele groups (*P* < 0.01) than that detected in fertile men; the percentage of sperm necrosis was significantly increased in the leukocytospermia group (*P* < 0.001) versus fertile men (Table 1).  Table 2 shows the comparisons of resistin level, MDA concentration, GSH/GSSG ratio, and CAT activity among the considered groups. Resistin values were significantly higher in the leukocytospermia group (*P* < 0.001) and the varicocele group (*P* < 0.01) than that measured in the control group. The MDA concentrations were significantly increased in both infertile groups (*P* < 0.001) compared to controls and also in the leukocytospermia group versus the varicocele group (*P* < 0.05). The GSH/GSSG ratio was significantly higher in fertile controls than the leukocytospermia group (*P* < 0.05) and the varicocele group (*P* < 0.001) and in the leukocytospermia group versus the varicocele group (*P* < 0.05). Both the leukocytospermia and varicocele groups showed higher values of CAT activities (*P* < 0.001) than that detected in the control group.

The correlations between all considered variables calculated in the 53 study participants are reported in Table 3. Sperm concentration, motility, and normal morphology were positively correlated to each other (*P* < 0.001). Sperm concentration positively correlated with GSH/GSSG ratio (*P* < 0.001) and negatively with necrosis (*P* < 0.001), resistin (*P* < 0.05), MDA concentration (*P* < 0.01), and CAT activity (*P* < 0.001). Sperm motility showed positive correlations with GSH/GSSG (*P* < 0.001) and negative correlations with apoptosis (*P* < 0.001), necrosis (*P* < 0.001), resistin (*P* < 0.05), MDA concentration (*P* < 0.001), and CAT activity (*P* < 0.001). The percentage of sperm with normal morphology showed a positive correlation with GSH/GSSG ratio (*P* < 0.001) and negative correlations with sperm apoptosis and necrosis (*P* < 0.001), resistin (*P* < 0.001), MDA concentration (*P* < 0.001), and CAT activity (*P* < 0.001). Sperm apoptosis was positively correlated with necrosis (*P* < 0.05), resistin (*P* < 0.01), MDA concentration (*P* < 0.05), and CAT activity (*P* < 0.01). Sperm necrosis displayed positive correlations with resistin (*P* < 0.001), MDA concentration (*P* < 0.01), and CAT activity (*P* < 0.05). Resistin was positively correlated with MDA concentration (*P* < 0.01) and CAT activity (*P* < 0.001) and negatively with GSH/GSSG ratio (*P* < 0.05). CAT activity was positively correlated with MDA concentration (*P* < 0.01) and negatively with GSH/GSSG ratio (*P* < 0.001).

## 4. Discussion

Here, we reported a relationship between resistin, LPO, and redox imbalance in human semen. Resistin is an adipokine able to trigger proinflammatory state in vitro and in vivo and to play a role in inducing the expression of other cytokines [11], so it may be considered an inflammatory biomarker [28]. Evidence for a crosstalk between adipokines, including resistin, inflammation, and redox imbalance, has been provided in several tissues and in different pathological conditions [1, 3, 29, 30].

Since in a previous research a significant relationship between resistin and proinflammatory cytokines was demonstrated in human semen samples [14], we decided to explore the potential relationships between resistin, LPO, CAT activity, and GSH/GSSG ratio in human semen. It is well known that oxidative stress plays a detrimental role on sperm function damaging membranes by LPO and causing mitochondrial and nuclear DNA fragmentation. Spermatozoa are vulnerable to ROS attack since their membranes are particularly rich in polyunsaturated fatty acids. LPO induces a loss of membrane properties such as membrane permeability and membrane potential and generally affects the cellular integrity [31]. LPO generates highly reactive lipid peroxidation products such as malondialdehyde (MDA), 4-hydroxynonenal, and isoprostanes, which all represent the best products to study such a process [32]. To protect sperm from the damages induced by oxidants, human semen is equipped with nonenzymatic and enzymatic antioxidants [33]. The reduced GSH is a nonenzymatic antioxidant able to react with lipid peroxides, protecting sperm plasma membranes [34]. The ratio GSH/GSSG was utilized in this study because it offers a simple representation of oxidative stress [35]. The enzyme CAT is able to decompose hydrogen peroxide to water and oxygen; thus, CAT is able to protect cells from oxidative damages caused by ROS [36].

Leukocytospermia and varicocele are both conditions associated with oxidative stress, a consequence of inflammatory situations [20, 37–41]. In addition, it is known that macrophages and neutrophils are both major sources of resistin involved in the inflammatory pathway [8, 11]; thus, leukocytospermia appears to be an interesting pathology to study. Likewise, it is reported that varicocele induces inflammation and, consequently, impairs spermatogenesis [42]. Recently, Ghandehari-Alavijeh et al. [43] suggested a relevant involvement of the hypoxia pathway in the etiology of varicocele leading to decrease sperm quality and DNA integrity. It is known that the presence of this pathology increases the levels of oxidants and reduces those of antioxidants [40].

In a previous paper, we demonstrated that semen resistin was increased in patients with leukocytospermia and smoking habit and also in infertile patients when we compare them with control subjects with unknown reproductive potential [14]. In this study, the patient's selection was more stringent and we selected infertile patients with abacterial leukocytospermia, infertile patients with varicocele, and a group of fertile patients used as control.

The data from this research showed that infertile patients affected by leukocytospermia and varicocele had increased levels of resistin with respect to fertile men. The impairment of redox status of both pathologies is highlighted by the decrement of GSH/GSSG ratio and the increase of MDA in both groups of infertile patients. Fraczek et al. [38] reported a high MDA concentration in sperm lysates in the presence of leukocytes, and Ni et al. [44] observed an increased MDA concentration in normozoospermic men with clinical varicocele and in infertile astheno/oligozoospermic patients with clinical varicocele. It is noteworthy that, in our study, MDA was significantly increased in the leukocytospermia group compared to the varicocele group, indicating that leukocytes, known to be one of the major sources of ROS and to stimulate sperm to produce ROS [45], can induce the oxidative stress. The GSH/GSSG ratio is decreased in both groups of infertile patients, particularly in varicocele patients, where following the unsuccessful attempt to counterbalance the excess of ROS, redox imbalance is predominant. Semen CAT activity is increased in the presence of leukocytospermia and varicocele, suggesting a sort of “chronic oxidative stress.” In some cases, enhanced ROS amounts cannot be neutralized even in the presence of increased expression of antioxidants and related enzymes. For this reason, enhanced ROS levels can be stabilized and lead to modifications of several cell components, perturbing homeostasis [46]. This observation fits with our results as the high MDA levels detected in the leukocytospermia and varicocele groups indicated that the increased CAT activity was not completely effective to counteract the excess of hydrogen peroxide which led to LPO and cell damage. Obviously, to draw final conclusions, additional redox indicators, other than CAT activity and GSH/GSSG ratio, should be evaluated at the same time.

It is well known that, in human semen, resistin is positively correlated with proinflammatory cytokines [14, 15]. In this research, the association between resistin, LPO, CAT activity, and GSH/GSSG ratio is a novel finding. Resistin was negatively correlated with semen parameters and GSH/GSSG ratio and positively with indices of sperm death, apoptosis, and necrosis and with MDA and CAT activities. These data confirm the relationship between resistin, inflammatory processes, and the relative redox imbalance. We then hypothesized that the presence of leukocytes in semen and varicocele could cause an increase in resistin (and other cytokines) levels concomitant with a redox imbalance able to influence semen quality: sperm motility was decreased and apoptosis was enhanced in both pathologies as also observed by other authors [47]. Sperm necrosis was particularly increased in the leukocytospermia group, in which the level of MDA was significantly higher than that observed in varicocele and fertile samples.

Very few data are available in the literature on the mechanism of action through which resistin can be involved in an inflammatory pathway in the human male reproductive system. Three receptors for resistin have been proposed: decorin, adenylyl cyclase-associated protein 1, and Toll-like receptor 4 (TLR4) [48]. Different experimental and clinical researches identified the TLR4 signaling pathways activated by resistin as the molecular mechanism that links insulin resistance and obesity [49]. Interestingly, TLR2 and TLR4 are both localized in the acrosomal and tail regions of human sperm [50]. Recently, Hagan et al. [51] found that TLR2 and TLR4 are both upregulated in the seminal plasma of patients with leukocytospermia. These data could represent the link between resistin and inflammation in human semen.

## 5. Conclusions

The results of this study indicate that resistin levels are increased in infertile patients with leukocytospermia and with varicocele and are correlated with impaired sperm quality, LPO, and semen redox imbalance. It should be interesting to test if antioxidant supplementation, in the presence of different pathologies, is able to influence the resistin level in the male reproductive system.

## Figures and Tables

**Table 1:** Semen parameters (means ± standard error) of infertile men are classified into 2 groups according to clinical diagnoses and fertile controls.

Variables	Leukocytospermia L	Varicocele V	Fertile men F	HDS Tukey post hoc test (*P* value)	Games-Howell post hoc test (*P* value)
Volume (ml)	3.51 ± 0.26	3.83 ± 0.35	3.58 ± 0.24	—	—
Sperm/ml ×10^6^	56.07 ± 7.30	69.34 ± 7.15	166.86 ± 19.60	—	F *vs.* L^∗∗∗^ F *vs.* V^∗∗∗^
Motility %	30.52 ± 2.33	26.65 ± 2.84	51.76 ± 1.15	F *vs.* L^∗∗∗^ F *vs.* V^∗∗∗^	
Normal morphology %	8.74 ± 0.34	8.06 ± 0.46	17.06 ± 0.51	F *vs.* L^∗∗∗^ F *vs.* V^∗∗∗^	
Apoptosis %	13.63 ± 1.44	13.82 ± 1.69	7.47 ± 0.99	F *vs.* L^∗∗^ F *vs.* V^∗∗^	
Necrosis %	20.10 ± 1.31	15.29 ± 2.15	10.64 ± 1.15	F *vs.* L^∗∗∗^	

Legend: volume (ml); sperm/ml ×10^6^ (number of sperm/ml); motility % (percentage of rapid and slow progressive sperm motility); normal morphology % (percentage of sperm with normal morphology assessed with Papanicolaou staining); apoptosis % (percentage of sperm apoptosis assessed with AnV/PI assay); necrosis % (percentage of sperm necrosis assessed with AnV/PI assay). ^∗∗^*P* < 0.01; ^∗∗∗^*P* < 0.001.

**Table 2:** Resistin and stress oxidative parameters (means ± standard error) of infertile men classified into 2 groups according to clinical diagnoses and fertile controls.

Variables	Leukocytospermia L	Varicocele V	Fertile men F	HDS Tukey post hoc test (*P* value)	Games-Howell post hoc test (*P* value)
Resistin (ng/ml)	3.13 ± 0.46	2.66 ± 0.49	0.84 ± 0.25		F *vs*. L^∗∗∗^ F *vs*. V^∗∗^
MDA (nmoli/ml)	6.87± 1.25	3.56± 0.61	0.56± 0.05	—	F *vs* L^∗∗∗^ F *vs*. V^∗∗∗^ L *vs*. V^∗^
GSH/GSSH	11.20 ± 0.97	8.12 ± 0.77	14.49 ± 0.66	F *vs*. L^∗^ F *vs*. V^∗∗∗^ L *vs*. V^∗^	
CAT (nmoli/min/mg of proteins)	9.61 ± 0.91	8.21 ± 0.73	3.02 ± 0.40	—	F *vs*. L^∗∗∗^ F *vs*. V^∗∗∗^

Legend: resistin (ng/ml); MDA (malondialdehyde; nmoli/ml); GSH/GSSG (reduced glutathione/oxidized glutathione; nmol/mg of protein); CAT (catalase activity; nmoli/min/mg of protein). ^∗^*P* < 0.05; ^∗∗^*P* < 0.01; ^∗∗∗^*P* < 0.001.

**Table 3:** Correlations (rho Spearman's coefficient) between all considered variables in 53 individuals.

	Sperm/ml ×10^6^	Motility %	Normal morphology %	Apoptosis %	Necrosis %	Resistin	MDA	GSH/GSSG	CAT
Sperm/ml ×10^6^	**1**								
Motility %	**0.674** ^∗∗∗^	**1**							
Normal morphology %	**0.677** ^∗∗∗^	**0.733** ^∗∗∗^	**1**						
Apoptosis %	**-0.255**	**-0.444** ^∗∗∗^	**-0.450** ^∗∗∗^	**1**					
Necrosis %	**-0.432** ^∗∗∗^	**-0.506** ^∗∗∗^	**-0.455** ^∗∗∗^	**0.262** ^∗^	**1**				
Resistin	**-0.312** ^∗^	**-0.316** ^∗^	**-0.445** ^∗∗∗^	**0.378** ^∗∗^	**0.470** ^∗∗∗^	**1**			
MDA	**-0.355** ^∗∗^	**-0.471** ^∗∗∗^	**-0.444** ^∗∗∗^	**0.318** ^∗^	**0.413** ^∗∗^	**0.416** ^∗∗^	**1**		
GSH/GSSG	**0.463** ^∗∗∗^	**0.497** ^∗∗∗^	**0.591** ^∗∗∗^	**-0.169**	**-0.216**	**-0.293** ^∗^	**-0.124**	**1**	
CAT	**-0.671** ^∗∗∗^	**-0.464** ^∗∗∗^	**-0.605** ^∗∗∗^	**0.390** ^∗∗^	**0.330** ^∗^	**0.453** ^∗∗∗^	**0.411** ^∗∗^	**-0.507** ^∗∗∗^	**1**

Legend: sperm/ml ×10^6^ (number of sperm/ml); motility % (percentage of rapid and slow progressive sperm motility); normal morphology % (percentage of sperm with normal morphology assessed with Papanicolaou staining); apoptosis % (percentage of sperm apoptosis assessed with AnV/PI assay); necrosis % (percentage of sperm necrosis assessed with AnV/PI assay); resistin (ng/ml); MDA (malondialdehyde; nmoli/ml); GSH/GSSG (reduced glutathione/oxidized glutathione; nmoli/mg of protein); CAT (catalase activity; nmoli/min/mg of protein). ^∗^*P* < 0.05; ^∗∗^*P* < 0.01; ^∗∗∗^*P* < 0.001.

## Data Availability

The data are reported in the paper.
